# Evaluation of a Novel Content-Based Image Retrieval System for the Differentiation of Interstitial Lung Diseases in CT Examinations

**DOI:** 10.3390/diagnostics11112114

**Published:** 2021-11-15

**Authors:** Tobias Pogarell, Nadine Bayerl, Matthias Wetzl, Jan-Peter Roth, Christoph Speier, Alexander Cavallaro, Michael Uder, Peter Dankerl

**Affiliations:** 1 Department of Radiology, University Hospital Erlangen, Maximiliansplatz 3, 91054 Erlangen, Germany; nadine.bayerl@uk-erlangen.de (N.B.); matthias.wetzl@uk-erlangen.de (M.W.); jan-peter.roth@uk-erlangen.de (J.-P.R.); alexander.cavallaro@uk-erlangen.de (A.C.); michael.uder@uk-erlangen.de (M.U.); peter.dankerl@uk-erlangen.de (P.D.); 2 Medizinische Fakultät, Friedrich-Alexander-Universität Erlangen-Nürnberg (FAU), Krankenhausstraße 12, 91054 Erlangen, Germany; 3 Siemens Healthcare GmBH, Siemensstraße 3, 91301 Forchheim, Germany; christoph.speier@siemens-healthineers.com

**Keywords:** content-based image retrieval, computer-aided diagnosis, interstitial lung disease, computed tomography

## Abstract

To evaluate the reader’s diagnostic performance against the ground truth with and without the help of a novel content-based image retrieval system (CBIR) that retrieves images with similar CT patterns from a database of 79 different interstitial lung diseases. We evaluated three novice readers’ and three resident physicians‘ (with at least three years of experience) diagnostic performance evaluating 50 different CTs featuring 10 different patterns (e.g., honeycombing, tree-in bud, ground glass, bronchiectasis, etc.) and 24 different diseases (sarcoidosis, UIP, NSIP, Aspergillosis, COVID-19 pneumonia etc.). The participants read the cases first without assistance (and without feedback regarding correctness), and with a 2-month interval in a random order with the assistance of the novel CBIR. To invoke the CBIR, a ROI is placed into the pathologic pattern by the reader and the system retrieves diseases with similar patterns. To further narrow the differential diagnosis, the readers can consult an integrated textbook and have the possibility of selecting high-level semantic features representing clinical information (chronic, infectious, smoking status, etc.). We analyzed readers’ accuracy without and with CBIR assistance and further tested the hypothesis that the CBIR would help to improve diagnostic performance utilizing Wilcoxon signed rank test. The novice readers demonstrated an unassisted accuracy of 18/28/44%, and an assisted accuracy of 84/82/90%, respectively. The resident physicians demonstrated an unassisted accuracy of 56/56/70%, and an assisted accuracy of 94/90/96%, respectively. For each reader, as well as overall, Sign test demonstrated statistically significant (*p* < 0.01) difference between the unassisted and the assisted reads. For students and physicians, Chi²-test and Mann-Whitney-U test demonstrated statistically significant (*p* < 0.01) difference for unassisted reads and statistically insignificant (*p* > 0.01) difference for assisted reads. The evaluated CBIR relying on pattern analysis and featuring the option to filter the results of the CBIR by predominant characteristics of the diseases via selecting high-level semantic features helped to drastically improve novices’ and resident physicians’ accuracy in diagnosing interstitial lung diseases in CT.

## 1. Introduction

To date, myriads of radiological searches have one root—Radiopaedia. Therefore, most radiologists will have come along this dictionary’s definition of interstitial lung diseases as “an umbrella that embraces a broad spectrum of disorders that are characterized by diffuse cellular infiltrates in periacinar location” [1]. More than 200 various lung disorders [2] from acute diseases (e.g., acute exacerbation UIP [3]) to more chronic fibrotic illnesses (e.g., sarcoidosis [4]) and most recently Post COVID-19 fibrosis [5], are represented in the family of interstitial lung diseases, most of which are very rare [6]. They often present with overlapping pathological, radiological and clinical features [7] as “chronic, progressive fibrosing interstitial pneumonia of unknown cause (…) characterized by progressive worsening of dyspnea and lung function” [8]. High-resolution CT (HRCT) plays a vital role in the diagnosis of interstitial lung diseases. For example, in the correct clinical context, when HRCT pattern is suggestive of usual interstitial pneumonia (UIP), surgical lung biopsy is not mandatory for a diagnosis of idiopathic pulmonary fibrosis (IPF) [9,10]. For the diagnosis and subsequent treatment of patients with interstitial lung disease (ILD) accurate pattern recognition on computed tomography (CT) images plays a vital role [11]. Correct and early diagnosis is crucial for deciding on the right treatment and presents a challenge, especially for novices. Therefore, advancements in computer-aided pattern recognition can improve the diagnostics of ILDs, make it accessible to non-academic centers and have the potential to cut down the costs spent on the social and healthcare aspects of the diseases [7]. Computer-aided diagnosis systems (CAD) (and CADs that utilize content-based image retrieval (CBIR)) have already been established in radiology such as in the detection of breast cancer in mammography or MRI [12,13,14], in the detection of lung metastases in MDCT images [15], or in the characterization of liver lesions in CT scans [16] just to name a few. With the recent emergence of artificial intelligence algorithms, likely to be one of the most promising advancements in the history of medicine [17], these have led to astonishing progress in image-recognition tasks and may someday even supersede human performance in complex tasks [18]. In fact, CADs have already been developed for the automatic classification of HRCT images [11]. In the present however, AI-like CAD systems are only used as a supplementary aid for the radiologists’ subjective image assessment [19]. Therefore, the performance of CADs is depending on the users’ knowledge and experience and has been shown to successfully assist even novice readers [20]. Regarding ILDs, while CADs have shown promising results such as good accuracy in detecting fibrosis on a quantitative level [21] and good performance in distinguishing between specific diseases such as UIP and non-specific interstitial pneumonia (NSIP) [22], current systems are usually limited in the number of different ILDs they comprise of and patterns they recognize. Given the challenges and implications of correctly diagnosing both common and rarer ILDs and identifying their underlying radiological pattern, we evaluated a novel CBIR for the differentiation of interstitial lung diseases that offers reference cases of 79 different lung diseases and featured the use of high-level semantic features.

Our goal was to compare the diagnostic performance of novice readers (students in their last year of training) and resident radiologists (at least three years of experience) with and without the assistance of the novel CBIR.

The underlying hypothesis was that the CBIR would assist in improving diagnostic performance of both groups while simultaneously narrowing the gap between the two.

## 2. Materials and Methods

This study was conducted in accordance with the guidelines of the Declaration of Helsinki and approved by the Ethics Committee of the University Hospital Erlangen under the approval number 258_18B. The Ethics Committee waived the written informed consent requirement.

We evaluated the performance of three novices (medical students in their final clinical year) and three radiology residents with at least three years of clinical experience in diagnosing interstitial lung diseases (ILDs) in CT examinations. Both cohorts evaluated the study population of ILD patients with and without the assistance of the commercially available content-based image retrieval system (CBIR) “ Similar Patient Search” (SPS) (Siemens Healthineers, Erlangen, Germany) which is embedded in the advanced visualization software *syngo*.via (Siemens Healthineers, Erlangen, Germany).

### 2.1. Patient Population

To create our study population, we filtered our RIS/PACS system for ILDs from September 2004–October 2020. We sorted for diagnoses represented in the evaluated CBIR. We selected 50 patients featuring 24 different diseases. To confirm the diagnosis of the written reports we gathered all clinical information about each patient out of the hospital information system to for instance check for blood workup (e.g., ACE values in sarcoidosis) or histology when available. We then manually selected representative examinations. To further validate the diagnosis in patients without histology a senior physician (with 11 years of experience) reevaluated these CTs together with all clinical information to define a ground truth. For the study cohort underlying diseases were chosen based on prevalence from common (e.g., UIP, NSIP) to very rare (e.g., Kartagener syndrome, or Swyer-James syndrome). In the end our study cohort consisted of: UIP/IPF (8 cases), NSIP (5), pulmonary emphysema/chronic obstructive pulmonary disease (5), cystic fibrosis (4), bronchiolitis (3), COVID-19 (3), pneumocystis pneumonia/atypical pneumonia (2), miliary tuberculosis (2), sarcoidosis (2), silicosis (2), pulmonary abscess (1), lobar pneumonia (1), aspergillosis (1), mycobacterial non-tuberculosis infection (1), obliterative bronchiolitis (1), pulmonary Goodpasture syndrome (1), granulomatosis with polyangiitis (1), Kartagener syndrome (1), chronic eosinophilic pneumonia (1), alveolarproteinosis (1), cryptogenic organizing pneumonia (1), Swyer-James syndrome (1), mucormycosis (1), lymphangioleiomyomatosis (1).

To further be able to evaluate not only specific disease, but radiological pattern as well, we assigned all 50 cases one of 10 different characteristic ILD patterns in HRCT: reticulation (7 cases), emphysema (7), honeycombing (6), bronchiectasis (6), consolidation (6), micro nodules (6), ground glass opacity (5), tree-in-bud (4), halo (2) and reversed halo (1). For the experimental reads the participants had to characterize the dominant pattern in each case and were given the option of presenting up to three differential diagnoses.

### 2.2. Content-Based Image Retrieval System

SPS (Siemens Healthineers, Erlangen, Germany) was used from within the advanced visualization software *syngo*.via (Siemens Healthineers, Erlangen, Germany). Here SPS can be triggered by drawing a ROI around pathological lung tissue in an axial lung CT.

The software displays images of cases similar to the outlined ROI and their corresponding diagnosis from a cloud database. The result-list additionally provides the typical imaging signs (provided by Thieme eRef (Georg Thieme Verlag KG, Stuttgart, Germany)) that are associated to each disease and can be filtered by tags which represent common characteristics of the diseases (chronic, infectious, smoking status, etc.) Furthermore, eRef reference content provided by the publisher Thieme can be accessed for each of the diseases containing detailed information about the disease’s definition, imaging signs, clinical findings, differential diagnosis, tips and pitfalls as well as selected references.

### 2.3. Training of the Novices

The three novices (medical students in their final clinical year) received a dedicated teaching to prepare them for the evaluation of ILDs prior to the first evaluation of cohort cases. Therefore, they were given a pictorial manual rehearsing the 10 different radiological patterns typically encountered in ILD patients: honeycombing, reticulation, ground-glass opacity, emphysema, micro-nodules, consolidation, bronchiectasis, tree-in-bud, halo and reversed-halo. The students were not allowed to use the manual during the test runs.

### 2.4. Case Evaluation Using Web-Based Presentation and Answer Sheet

**Figure 1:**
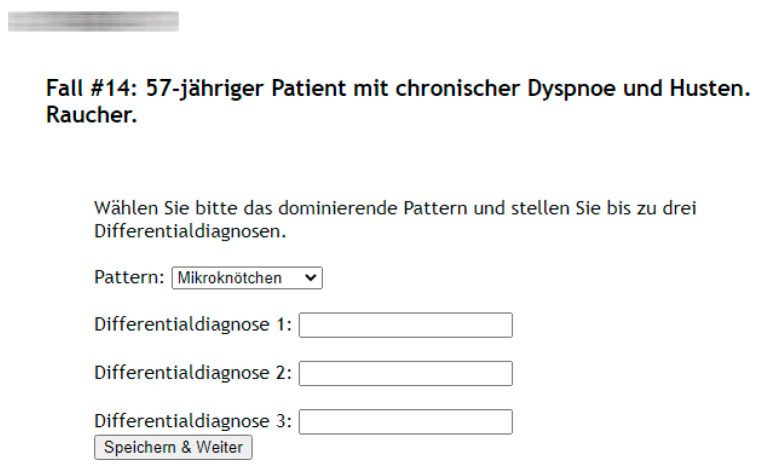
Example of a web-based case presentation and answer sheet featuring a short patient history consisting of age and sex as well as cardinal symptoms and relevant information (e.g., 57-year-old male with chronic dyspnea and coughing, smoker).

To document the study participants’ reading results we created a web-based case presentation and answer sheet in the German language. For each case it features a short patient history consisting of the age and sex as well as cardinal symptoms (e.g., dry cough and dyspnea) and relevant information such as if the patient is a smoker (Figure 1).

Together with this clinical information for each case the readers were given 5 mm transversal lung window CTs of the 50 patients. Thereby all answers of the participants were documented via the web-based answer-sheet. For the first evaluation of each case the readers had to decide on the dominant pathological pattern. Therefore, they utilized a drop-down menu consisting of the 10 patterns and up to three unique differential diagnoses in the form of free text inputs. The readers did not receive any feedback concerning the correctness of their answers.

**Figure 2:**
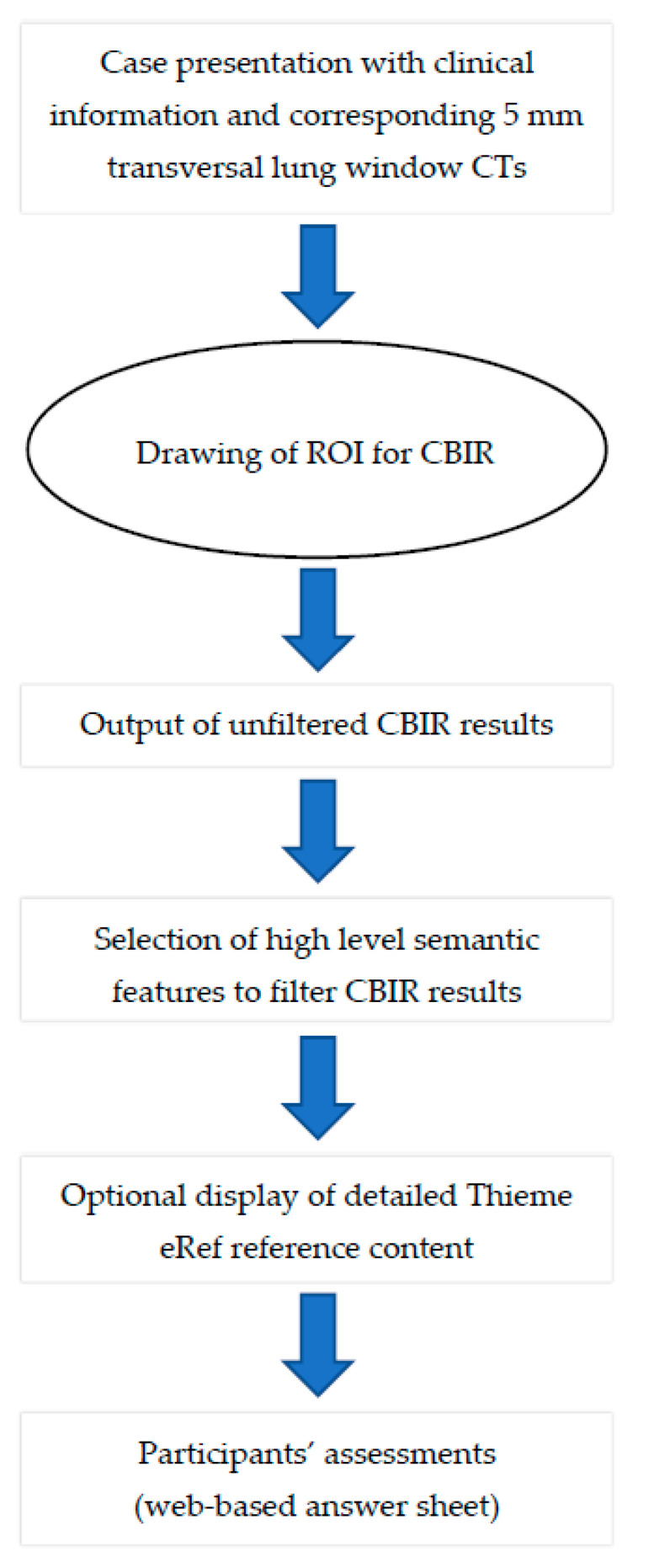
Workflow for each case presented to the participants.

**Figure 3:**
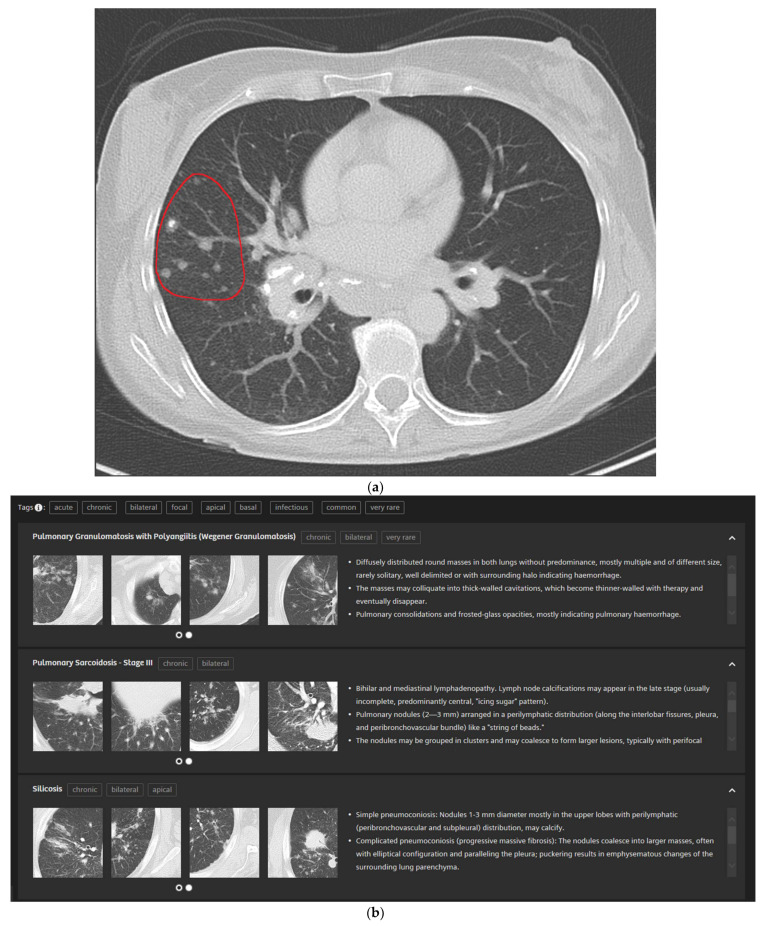

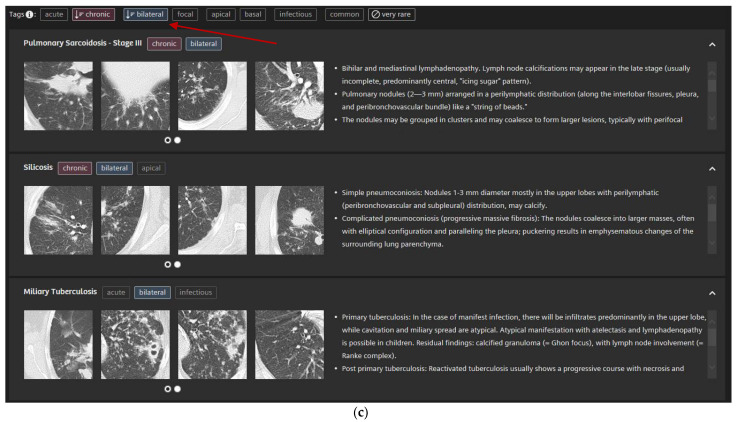
5 mm transversal lung window CT of a 51-year-old female patient with pulmonary sarcoidosis and corresponding CBIR user interface: Participants were shown the corresponding 5 mm transversal lung window CTs for each case. They then drew a ROI into the area with their supposed dominant pattern (**a**). Readers were then presented the CBIR results (**b**) and could select high-level semantic features (e.g., chronic, bilateral) to filter retrieval results (**c**).

Within a two-month interval and in a random order the readers reevaluated the 50 cases with the assistance of the CBIR according to the following procedure (Figure 2):

In detail, the participants drew a ROI into their perceived representative pattern in each case which was then analyzed by the CBIR (Figure 3a). The readers are then presented with the CBIR results (Figure 3b). To further improve the retrieval results the CBIR features the possibility to input clinical information in the form of high-level semantic features (e.g., smoking, infectious, chronic etc.) (Figure 3c).

### 2.5. Statistical Analysis

Accuracy was calculated based on the number of cases each participant correctly diagnosed in each test run divided by the overall number of cases. A diagnosis was counted as correct if at least one of the three possible entries of the participants matched the ground truth diagnosis.

To further investigate another layer of typical radiological workflow and the possibility to give multiple differential diagnoses we employed a weighting system (weighted accuracy, 0–150 points with a maximum of 3 points per case) that was calculated as following:

If the participant’s entry/entries on a given case didn’t contain the correct diagnosis the participant was awarded 0 points.

- Three entered differential diagnoses containing the correct answer were awarded 1 point.
- Two entered differential diagnoses containing the correct answer were awarded 2 points.
- A correct differential diagnosis while being the sole answer given was awarded 3 points.

Pattern Accuracy was calculated based on the number of patterns each participant correctly identified in each test run. A pattern was counted as correct if it matched the ground truth diagnosis.

SPSS 27 (IBM Corporation, Armonk, NY, USA) was used for the statistical analysis. The data was tested for normal distribution using the Shapiro-Wilk test, rejecting the null-hypothesis. The data was tested for statistically significant difference (*p* < 0.01) between assisted and unassisted reads using Sign test for accuracy and pattern accuracy and Wilcoxon signed-ranked test for weighted accuracy. The data was tested for statistically significant difference (*p* < 0.01) between students and physicians in assisted and unassisted reads using Chi²-test for accuracy and pattern accuracy and Mann-Whitney-U test for weighted accuracy.

## 3. Results

### 3.1. Accuracy

The mean accuracy of the students was 30.0% in the unassisted test runs and 85.3% in the CBIR-assisted test runs, respectively.

The mean accuracy of the radiology residents was 60.7% in the unassisted test runs and 93.3% in the CBIR-assisted test runs, respectively.

The difference in mean accuracy between the students and the residents proved to be significantly different (*p* < 0.01) in the unassisted test runs and not significantly different (*p* = 0.025) in the CBIR-assisted test runs (Please see Table 1 and Table 2 for individual results of students and residents, respectively).

### 3.2. Weighted Accuracy

The mean weighted accuracy of the students was 26.0% in the unassisted test runs and 81.3% in the CBIR-assisted test runs, respectively.

The mean weighted accuracy of the radiology residents was 56.9% in the unassisted test runs and 89.3% in the CBIR-assisted test runs, respectively.

The difference in mean weighted accuracy between students and residents proved to be significantly different (*p* < 0.01) in the unassisted test runs and not significantly different (*p* = 0.065) in the CBIR-assisted test runs (Please see Table 3 and Table 4 for individual results of students and residents, respectively).

### 3.3. Pattern Accuracy

The mean pattern accuracy of the students was 60.0% in the unassisted test runs and 83.3% in the CBIR-assisted test runs, respectively.

The mean pattern accuracy of the radiology residents was 76.0% in the unassisted test runs and 85.3% in the CBIR-assisted test runs, respectively.

The difference in mean pattern accuracy between students and radiology residents proved to be significantly different (*p* < 0.01) in the unassisted test runs and not significantly different (*p* = 0.634) in the CBIR-assisted test runs (Please see Table 5 and Table 6 for individual results of students and residents, respectively).

## 4. Discussion

As demonstrated in our study the novel CBIR significantly helped to improve novices’ as well as resident physicians’ accuracy in diagnosing ILDs in CT. In addition, the mean improvement in accuracy of the novice readers with CBIR assistance was substantial in a way that they outperformed resident physicians’ unassisted results. Consequently, statistical insignificance when comparing CBIR assisted results of novices and resident physicians shows that much less experience is needed to accurately diagnose most ILDs in CT using the novel presented CBIR. One could argue that the novice readers’ increase in performance during the second read was some kind of learning effect. However, we tried to exclude this throughout our study design firstly by not giving them feedback during their first experimental read to what the correct answers were, and secondly by displaying the CTs in random order with a two-month interval.

Radiology is often on the forefront for technological advancements in medicine and pattern recognition in conjunction with image interpretation are certainly areas surrounded with the most excitement regarding future advancements [23]. AI can already achieve detection of, e.g., pneumonia comparable to radiologists as shown by Rajpurkar et al. [24], or accurately detect pulmonary nodules [25]. However, fully automatic and accurate diagnosing of complex diseases such as ILDs is most likely still years away. In the meantime, efforts should be made to ease the process and lessen the experience needed in finding the correct diagnosis. Especially for novices and inexperienced radiologists, correctly interpreting the radiological patterns present in ILDs and consequently coming up with fitting differential diagnoses is a big challenge. Furthermore, this is very time consuming and can sometimes be somewhat impossible as some ILDs are very rarely encountered in daily workflow.

Typically, in our study when presented with a pattern or disease the readers weren’t familiar with or hadn’t encountered yet, all they could ultimately do without the assistance of the CBIR is make an (un-)educated guess. This was especially the case for the novices. In contrast to that we found that, when using the in this work evaluated novel CBIR and given a few differential diagnoses that could then be evaluated by comparing images and with the comprehensive information about each disease included in the CBIR (Figure 3), participants would come to the correct conclusion most of the time.

In 2002 the American Thoracic Society/European Respiratory Society in a joint statement proposed a multidisciplinary approach centered around including pathologists, radiologists and clinicians in the process of diagnosing ILDs [26,27]. Since HRCT is key in the detection and diagnosis of ILDs and HRCT often acts as the first gauge of the status quo in a patient‘s lung it is crucial that we, as radiologists, correctly detect pathological patterns as early as possible and point the diagnostic process in the right direction. This is especially relevant in non-academic centers where multidisciplinary approach might not be possible and experience with ILDs might be shallow.

We believe that the presented novel CBIR can help speed up the process of diagnosing ILDs and therefore improve patient treatment, ultimately ameliorating patient outcome and reducing costs to the healthcare system.

A strength of our study was the inclusion of a wide variety of diseases and radiological patterns and the inclusion of novices as well as experienced resident radiologists to quantify the effect of the evaluated CBIR on the experience required to diagnose ILDs. However, our study has some limitations. First, we didn’t measure the time each participant needed to finish each test run, so we didn’t quantify if using the evaluated CBIR positively or negatively impacted radiological workflow timewise.

Second, in cases without histologically confirmed diagnoses our ground truth consisted of an expert radiologist’s evaluation containing all available clinical information as well as disease development over time.

## 5. Conclusions

The evaluated CBIR relying on pattern analysis and featuring the option of inputting clinical information as high-level semantic features, drastically helped improve novices’ and resident physicians’ accuracy in diagnosing interstitial lung diseases in CT. Moreover, Statistical insignificance for the evaluated CBIR-assisted test runs between novices against the radiology residents showed to narrow the gap in experience needed to correctly diagnose ILDs in CT.

## Figures and Tables

**Table 1:** Results of the students’ accuracy. All students’ accuracies in the unassisted test runs proved to be significantly different (*p* < 0.01) from the CBIR-assisted test runs.

	Student 1	Student 2	Student 3
Number of Cases	50	50	50
Correctly diagnosed unassisted	9	14	22
Accuracy unassisted	18.0%	28.0%	44.0%
Corrrectly diagnosed CIBR-assisted	42	41	45
Accuracy assisted	84.0%	82.0%	90.0%

**Table 2:** Results of the residents’ accuracy. All residents’ accuracies in the unassisted test runs proved to be significantly different (*p* < 0.01) from the CBIR-assisted test runs.

	Resident 1	Resident 2	Resident 3
Number of Cases	50	50	50
Correctly diagnosed unassisted	28	28	35
Accuracy unassisted	56.0%	56.0%	70.0%
Corrrectly diagnosed CBIR-assisted	47	45	48
Accuracy CBIR-assisted	94.0%	90.0%	96.0%

**Table 3:** Results of the students’ weighted accuracy. Point scores are out of a maximum of 150. All students’ weighted accuracies in the unassisted test runs proved to be significantly different (*p* < 0.01) from the CBIR-assisted test runs.

	Student 1	Student 2	Student 3
Points score unassisted	25	37	66
Weighted accuracy unassisted	16.7%	17.3%	44.0%
Points score CBIR-assisted	122	115	129
Weighted accuracy CBIR-assisted	81.3%	76.7%	86.0%

**Table 4:** Results of the residents’ weighted accuracy. Point scores are out of a maximum of 150. All residents’ weighted accuracies in the unassisted test runs proved to be significantly different (*p* < 0.01) from the CBIR-assisted test runs.

	Resident 1	Resident 2	Resident 3
Points score unassisted	81	76	99
Weighted accuracy unassisted	54.0%	50.7%	66.0%
Points score CBIR-assisted	138	123	141
Weighted accuracy CBIR-assisted	92.0%	82.0%	94.0%

**Table 5:** Results of the students’ pattern accuracy. Student 1’s and Student 2’s pattern accuracy in the unassisted test runs proved to be significantly different (*p* < 0.01) from the CBIR-assisted test runs, while Student 3’s was not significantly different (*p* = 0.25).

	Student 1	Student 2	Student 3
Correctly determined patterns unassisted	26	26	38
Pattern accuracy unassisted	52.0%	52.0%	76.0%
Correctly determined patterns CBIR-assisted	42	42	41
Pattern accuracy CBIR-assisted	84.0%	84.0%	82.0%

**Table 6:** Results of the radiology residents’ pattern accuracy. All radiology residents’ pattern accuracy in the unassisted test runs proved to be not significantly different from the CBIR-assisted test runs (*p* = 0.016, *p* = 0.031 and *p* = 0.19, respectively).

	Resident 1	Resident 2	Resident 3
Correctly determined patterns unassisted	36	37	41
Pattern accuracy unassisted	72.0%	74.0%	82.0%
Correctly determined patterns CBIR-assisted	42	42	44
Pattern accuracy CBIR-assisted	84.0%	84.0%	88.0%

## Data Availability

Data available on request due to restrictions, e.g., privacy or ethical. The data presented in this study are available on request from the corresponding author. The data are not publicly available due to privacy.
